# P15. A genetic mouse model to identify the role of the immune adapter protein MyD88 in colorectal cancer

**DOI:** 10.1186/2051-1426-2-S2-P6

**Published:** 2014-03-12

**Authors:** A Holtorf, B Holzmann, KP Janssen

**Affiliations:** 1 Klinikum rechts der Isar, Chirurgische Klinik und Poliklinik, Munich, Germany

## Background

Pattern recognition receptors from the Toll-like receptor (TLR) family are pivotal components of innate immunity, and have been shown to contribute to colon cancer formation. However, the molecular and cellular mechanisms underlying TLR-signaling in colon cancer remain unclear. The adapter protein *Myeloid-differentiation factor 88* (MyD88) is shared between several TLRs and the Interleukin-1 receptor family. MyD88-deficiency protects mice from intestinal cancer formation in genetic models for colon cancer. The genetic mouse model presented here allows tissue-specific expression of MyD88, and thereby the dissection of the complex interaction between tumour and immune system during intestinal carcinogenesis.

## Material and methods

Insertion of an 'intron-gene-trap' flanked with loxP motifs into the first intron of the MyD88 gene locus leads to global inactivation of *myD88* expression (MyD88^LSL^), faithfully phenotyping a global gene knock-out. Tissue-specific re-expression of MyD88 in mice is mediated based on the Cre-recombinase. Breeding of MyD88^LSL^ mice with LysMCre or pvillin-Cre mice leads to tissue-specific excision of the 'intron-gene-trap', retaining endogenous regulation of gene expression. MyD88 expression and successful reconstitution of TLR-signaling was detected in either myeloid cells (MyD88^MYEL^) or intestinal epithelial cells (MyD88^IEC^). Subsequently, these animals were mated with Apc^1638N/+^ mice, an established genetic mouse model for human colon cancer.

## Results

Global MyD88 deficiency dramatically decreased tumour incidence and aggressiveness in Apc^1638N/+^ mice. Re-expression of MyD88 in intestinal epithelial cells only partially restored tumor formation. On the other hand, reconstitution of MyD88 expression in myeloid cells triggered tumour development virtually indistinguishable from parental Apc^1638N/+^ mice. Activation of the canonical Wnt signaling pathway, induced by loss of function of Apc, was independent of MyD88. In contrast, MyD88 expression was required for full activation of MAPK/ERK signaling in intestinal epithelial cells. Furthermore, our results suggest a pro-tumorigenic function for the pro-inflammatory cytokines IL-1beta and IL-6, which were produced in a MyD88-dependent fashion by myeloid cells.

## Conclusions

MyD88-mediated signaling has pro-tumorigenic effects in both IECs and in myeloid cells, but via different mechanisms. Moreover, MyD88 function in myeloid cells is crucial for intestinal tumour development, and its inhibition may form a promising therapeutic strategy.

